# A Concise History of the Principal Discoveries in Anatomy

**Published:** 1799-12

**Authors:** 


### *A Concife History of the Principal Difcoveries in Anatomy.*

[Continued from our laft Number, pp. 368 — 373.]

§ 21. HAVING given a brief account of that important difcovery, the circulation of the blood, we fhall now proceed to examine the leading opinions and difcoveries, which relate to particular branches of the arterial and venous fyftems. — With refpect to the courfe of the *aorta*, this artery, when proceeding from the heart, was, in the times of VESALIUS divided into the afcending and defcending; though the former is a nonnon-entity,* becauſe this artery branches into the carotids, and into the ſubclavian arteries in the arch itſelf. This error was, without oſtentation, firſt corrected by EUSTACHIUS;† and, afterwards, by FABRICIUS.‡ The ſkilful anatomiſts, BERENGAR and VESALIUS, were the firſt who controverted the erroneous opinion, that the carotid artery, at its entrance into the brain near the *glandula pituitaria cerebri*, forms a *reticular texture*. Mean time Veſalius himſelf had, conſiſtently with truth, admitted a connection and anaſtomoſes between the carotids and the vertebral arteries; from which circumſtance he explained the continuation of life, even after the carotids had been divided.|| And theſe anaſtomoſes, (of which the immortal Fallopius gave a maſterly deſcription, and added to them the anaſtomoſis with the baſilic artery) he conſidered as the true *rete mirabile* that deſerves the admiration of the obſerver, in a degree at leaſt equal to thoſe windings of the carotids in animals.¶ Columbus endeavoured to defend Galen againſt Veſalius, by applying the aſſertion of the former, reſpecting the carotid, to the vertebral artery; which, when entering the great foramen of the occiput, obviouſly forms four conſiderable turnings, beſides many anaſtomoſes with the carotid and baſilic arteries, as well as with the *arteria communicans*.** KOYTER indulged his ſpeculations for defending Galen ſtill farther, when he referred the explanations given by that ancient writer, reſpecting the *rete* of the carotids, to the ramification of the third (fifth) pair of nerves in the baſis of the brain.††

§ 22. VESALIUS had obſerved the alternate ſwelling and ſinking of the brain, during inſpiration and expiration. As he was unacquainted with the larger circulation of the blood, he could not explain this phenomenon in any other manner, than by conjecturing that the blood veſſels in the brain were of an arterial nature; while he believed, that the arteries emptied themſelves into the latter.‡‡ Although FALLOPIUS |||| and COLUMBUS ¶¶ diſcovered that the blood veſſels of the brain belong to the venous ſyſtem, yet the changes of the brain during reſpiration, which KOYTER *** likewiſe obſerved, could not be ſatisfactorily explained previous to the diſcovery of the circulation.

EUSTACHIUS

* *Veſal.* Exam. Obſerv. *Falloſi.* lib. iii. c. 12. p. 341.

† *Euſtach.* Tab. xv. fig. 2, 4, 6.

‡ De format. foet. p. 52. Tab. vi. fig. 15.

|| *Veſal.* Lib. iii. c. 12. p. 342.

¶ *Falloſi.* Obſerv. p. 400.

** *Collumb.* Lib. vii. p. 337.

†† *Coiter* Obſerv. p. 123.

‡‡ *Veſal.* Lib. iii. c. 14. p. 350.

|||| *Falloſi.* Inſtit. p. 458.

¶¶ Columb. Lib. viii. p. 349.

*** *Coiter.* Obſerv. p. 122.

Eustachius very accurately pointed out the origin of the *arteria ethmoidea anterior*, from the *arteria ophthalmica orbitalis*.*

The spinal artery, which derives its origin from the *arteria cerebralis profunda*, or from the vertebral artery, and which proceeds downwards along the *tunica arachnoidea* of the *medulla spinalis*, had been noticed by Berengar, as a white shining line;† and Stephanus was at a loss, whether to consider it as a nerve running parallel with the spinal marrow.‡ This mistake is very pardonable, for even modern anatomists have considered this vessel as a ligament.§ The posterior arteries of the ear were perhaps first represented by Guidi.|| Both Vesalius and Eustachius made inquiries relative to the progress of the subclavian and axillary arteries and veins: the former reproached Galen for his superficial investigation of the branches belonging to the deep axillary vein, and demonstrated the branches proceeding from this vessel, and communicating with the common cutaneous veins.¶ Eustachius, on the contrary, endeavoured to prove, that Galen had doubtless known these veins; and the former described the anastomoses of the basilic, cephalic, and median veins.** But it must be admitted, that Eustachius was not sufficiently acquainted with the division of the humeral artery; as he asserts, that the arteries of the radius and ulna, arise from the former only below the elbow; while it is certain, that these arteries frequently begin above the ancon; and his drawing of the axillary vein is not altogether a faithful copy from nature.††

The origin of the left gastro-epiploic artery, Vesalius pretty accurately deduced from the splenic artery.‡‡ His error, however, that the external jugular veins were larger and more capacious than the internal, was corrected with great accuracy by Fallopius, who proved the contrary.§§ Vesalius, and several other cotemporary anatomists, had erroneously derived the arteries of the penis from the vessical artery: Fallopius corrected this mistake also, by proving that those

* Ofs. exam. p. 172.

† Comment. in Mundin. f. 496. b.

§ *Haller* Element. Physiol vol. iv. p. 136.

|| *Vid.* lib. iii. tab. 27. fig. 1. (qq) p. 124.

¶ *Vesal.* lib. iii. c. 8. p. 329.

** *Eustach.* Opusc. p. 292.

†† Tab. xxvi. No. 1. (n.)

‡‡ Lib. v. c. 4. p. 423. fig. 2. (R.)

§§ *Vesal.* lib. iii. c. 7. p. 327.—*Fallop.* Observ. p. 397.

those vessels really originated from the *arteria pudenda & arteria pelvis*: the former, or the *pudenda communis*, he called *hypocystica*.*

Vesalius, the immortal father of modern anatomists, appeared at length with a discovery which excited great attention, in consequence of the incorrect notions then prevalent, with respect to the circulation of the blood in the veins. He demonstrated, that the vena sine pari, which arises from the intercostal muscles and the pleura, terminates only in the right vena cava; or, as it was then expressed, that it originates from the latter, and proceeds to the pleura. If, therefore, this membrane were affected, the blood may be emptied by the nearest canal, if in such a case the axillary vein of the right arm were opened; because this vessel arises from the vena cava, near the vena sine pari.† He refuted Galen, who had maintained, that the last mentioned vein uniformly empties itself into the vena cava, in the cavity of the pericardium. Sylvius, the adversary of Vesalius, could not produce any valid objections against occular demonstration; but, in order to save the credit of his favourite Galen, he made use of his usual, though absurd evasion, that the human body, in ancient times, had been larger, consequently the cavity of the thorax had also been longer.‡ Eustachius, however, inquired more minutely into this subject, and made interesting remarks on the anastomoses of the vena sine pari with the renal veins, the truth of which was farther confirmed by Fallopius: § he also commented with ability on the occasionally double formation of this vein; || on the *hemiazyga*, ¶ and on the division of the vena sine pari near the eighth and ninth ribs.** He admitted that the azygos does not uniformly empty itself into the vena cava, within the pericardium; but maintained, that this efflux takes place *in the vicinity of* the pericardium.†† Lastly, Arantius likewise observed the anastomoses of the vena sine pari with the intercostal and axillary veins.‡‡

* *Fallop.* Observ. p. 419.

† *Vesal.* Epistol de usu radic. Chyn. p. 641.—*Id.* de corp. *human fabric.* lib. iii. c. 7. p. 323. (edit. *Albin.* fol. L. B. 1725.) But Vesalius had even, in the year 1539, written a particular epistle or treatise on this subject.

‡ *Sylv.* vesan. calumn. depuls. p. 98.—Comp. *Putei* apolog. f. 137. 6.

§ *Eustach.* de vena sine pari, p. 103, 109, 110.—*Fallop.* Instit. p. 448.—Comp. *Morgagni* adversar. anat. vol. v p. 86.

|| *Eustach.* Ibid. p. 279.

¶ *Ibid.* p. 275.

** *Ibid.* p. 290.

†† *Ibid.* p 244.

‡‡ *Arant.* Observ. c. 32. p. 90.

§ 23. The first trace of the knowledge of the *lymphatic vessels*, we find in HEROPHILUS ;* but this discovery was very little cultivated in a century, which produced so many eminent anatomists; and the doctrine of the lacteals and lymphatics made less progress, comparatively, than any other branch of anatomy. MASSA observed ducts proceeding upwards from the mouth of the renal vessels, so early as the year 1532; and these ducts, probably, were lymphatics.† FALLOPIUS still more plainly discovered canals, which proceeded from the surface of the liver to the pancreas, and were filled with a yellowish humour.‡ At length, EUSTACHIUS discovered, in horses, the principal trunk of the lacteals, or the *ductus thoracicus*.§ "From the inner part of the subclavian vein," says he, "there extends in these animals, downwards, a large vessel, the orifice of which is closed by a semilunar valve. This duct is white, and contains a watery humour. Near its origin, it branches into two divisions, which afterwards unite; and, without forming any other branches, the principal trunk proceeds on the left side of the dorsal vertebræ, through the diaphragm, to the middle of the lumbar region, where it dilates considerably, surrounds the great artery, and terminates in a manner hitherto unknown to me." Our ancestors had advanced thus far in the knowledge of the lymphatics, at the conclusion of the sixteenth century.

[*Want of room, occasioned by the influx of many valuable original communications, has obliged us to limit the extent of foreign articles accordingly. Hence the reader will, doubtless, excuse us for continuing this comprehensive and important article, through the whole of the next volume. The following branches of the History of Anatomy remain, therefore, to be detailed in future Numbers of this work, viz. Splanchnology—Organs of Generation—Structure of the Brain,—and Distribution of the Nerves.*]

---

* *Galen* de usu part. lib. iv. p. 417.

† *Sylv.* Isagog. f. 32. b.

‡ *Falloп.* Observ. p. 395.

§ *Eustach*, de vena sine pari. p. 280.

HINTS

